# Protective factors associated with resilience among unaccompanied refugee minors after settling in Norway: a matched cross-sectional study

**DOI:** 10.1007/s00787-023-02364-9

**Published:** 2024-01-29

**Authors:** Oda Marie Heimli, Ingrid Kvestad, Tormod Bøe, Nawar Sayyad, Sondre Aasen Nilsen, Sølve Randal, Kristin Gärtner Askeland

**Affiliations:** 1 RKBU Vest, NORCE Norwegian Research Centre, Bergen, Norway; 2 Department of Psychosocial Science, Faculty of Psychology, University of Bergen, Bergen, Norway; 3 Department of Health Promotion and Development, Faculty of Psychology, University of Bergen, Bergen, Norway; 4 Child Welfare Services for Unaccompanied Refugee Minors, Bergen Municipality, Bergen, Norway

**Keywords:** Unaccompanied refugee minors, Resilience, READ, Child welfare, Adolescents

## Abstract

This study aimed to examine protective factors associated with resilience among unaccompanied refugee minors in comparison to their Norwegian peers and to examine associations between resilience factors and characteristics related to positive outcomes among unaccompanied minors. Data stem from the *Pathways to Independence study* conducted in Bergen municipality, Norway in 2018–2019 where 81 unaccompanied minors aged 15–20 participated (83.3% male; 80% response rate). An age- and sex-matched control group of 324 adolescents was retrieved from the youth@hordaland study conducted in Norway in 2012. Resilience factors were assessed by the Resilience Scale for Adolescents. Unaccompanied minors reported lower scores on Goal Orientation (*d* = 0.4), Social Competence (*d* = 0.4), and Social Support (*d* = 0.7) compared to Norwegian adolescents. Being male was associated with lower scores on Goal Orientation (standardized mean difference [SMD] = − 0.9) and Social Support (SMD = − 0.9) among unaccompanied minors, while being in frequent contact with family in the home country was associated with higher scores on all resilience factors (SMD range = 0.6–1.1). The number of leisure activities was associated with Social Competence (SMD = 0.22). There were no significant associations between the resilience factors and amount of support in the living arrangements or contact with the child welfare services. Unaccompanied minors reported fewer resilience factors compared to Norwegian adolescents, indicating that they may have different needs compared to other adolescents. Our study also suggests that frequent contact with family in the home country may be important to bolster positive development for unaccompanied minors after settlement.

## Introduction

Unaccompanied refugee minors (unaccompanied minors) refer to asylum seekers who arrive in the host country before they turn 18, without a parent or legal guardian who has been granted a residence permit. In Norway, 10,118 persons, 84% males, obtained residence permits as unaccompanied minors between 1996 and 2020 [1]. The majority arriving in Norway in this period originated from Afghanistan, Eritrea, Syria, and Somalia. While unaccompanied minors are heterogeneous regarding gender, age, ethnicity, and religious background [2], they have often been exposed to traumatic experiences in their home country and during the flight [3]. It is estimated that 96% of unaccompanied minors in Norway have experienced at least one traumatic incident [4]. Still, it is suggested that 60% of unaccompanied minors cope well in the Norwegian society [5].

Resilience refers to the process wherein an individual copes well and has a better outcome than expected after risk exposure [6–8]. Notably, positive development in itself is insufficient to establish resilience; there must be a current or past risk with a known potential to disrupt development [8]. Positive development among unaccompanied minors could be considered a result of resilience due to their known history of risk exposure [5].

When studying resilience, the focus is often on the protective factors thought to underlie the process of resilience. These factors are multidimensional and often divided into individual, family, and community qualities [7]. Known protective factors are individual characteristics such as temperament and intelligence, socialization practices within the family that build trust and autonomy, and external support systems [9]. Investigating resilience among unaccompanied minors using comprehensive measures covering all these domains could give a broad overview of different strengths that can contribute to coping and positive development.

In general, research on the development of unaccompanied minors tends to focus on their emotional difficulties due to separation and previous trauma [10–15] and less on coping mechanisms and resilience [2]. Although few studies have investigated protective factors specifically related to resilience, some common characteristics have been associated with better outcomes among unaccompanied minors despite their different migratory experiences and cultural contexts [16]. Several studies have investigated demographic characteristics such as age, gender, region of origin, time spent in the host country, and living situation as predictors for mental health problems. A literature review suggests gender as a consistent predictor of psychological distress among unaccompanied minors [17] where female gender has been identified as a risk factor for internalizing symptoms, depression, traumatic stress symptoms, and stressful life events [5, 10–12, 18–24]. Reframing this in terms of protection, it suggests that being male is protective against the development of mental health problems among unaccompanied minors. Regarding age, results vary widely between studies and a review concluded that age is not a distinct predictor for psychological distress [17]. Similarly, region of origin does not seem to be an important predictor for mental health problems [12, 14, 15, 18, 25, 26]. Further, time spent in the host country has neither been considered as a protective nor a risk factor for poor mental health among unaccompanied minors [10–12, 20, 23, 25–27].

In contrast, having a higher degree of support in the living arrangements in the host country seems to be protective [17]. Unaccompanied minors living in high support living arrangements, such as foster care reported lower traumatic symptoms, less depression, less internalizing symptoms, and less psychological distress [12, 18, 19, 22, 27]. Living in foster care and receiving high support thus seems to be protective against developing a wide variety of psychological difficulties.

Regarding individual factors, both cultural competence [5, 21] and language proficiency [14] have been found to protect against developing depression and post-traumatic stress disorder among unaccompanied minors. Further, higher scores on everyday resources have been related to lower levels of total mental health problems, internalizing behavior, externalizing behavior, and more specifically symptoms of depression [14]. A case study from the US has also suggested a positive outlook, healthy coping mechanisms, and religiosity as protective for unaccompanied minors [28].

Several studies suggest that contact with family members who remain abroad can protect against developing mental health problems [13, 17, 21, 29, 30]. A majority of unaccompanied minors stay in contact with their biological family abroad [30], and those who were in contact with their families abroad perceived high levels of support and considered the family their most important social support [21]. Furthermore, contact with family has been associated with higher scores in the personal protective factor cultural competence and social support from peers, which suggests it could have a greater impact on later functioning than merely the support it entails [21].

Social support from outside the family is also important, especially for unaccompanied minors with limited familial support [30]. Social Support in the post-flight period strengthened functional coping with stressful experiences and fostered mental health [30]. Further, social support can protect against developing poor mental health [17], more specifically depression and symptoms of anxiety [14, 21]. These findings are backed by studies including all refugee children, where a stable settlement, social support, and a sense of belonging promoted resilience and mental health functioning [16, 31].

Based on decades of research on protective factors underlying positive development across adversities, several measurements have been developed to examine protective factors associated with resilience among adolescents [32]. Only one cross-sectional study of 18 males and 1 female in Italy has used a comprehensive resilience measure when studying unaccompanied minors, the Child and Youth Resilience Measure (CYRM) [33]. Despite adequate resilience levels compared to a normative sample from a different study, the unaccompanied minors reported psychological difficulties such as depression, anxiety, and post-traumatic symptoms [33]. In another study of 160 refugees, the resilience factors measured by the CYRM were not associated with post-traumatic symptoms, indicating that resilience and psychological symptomatology co-existed [34]. This highlights the importance of using specially developed resilience measures, as they measure basic protective factors that influence several domains of life, not just mental health.

### Aims

This study aimed to examine self-reported protective factors, hereby referred to as resilience factors, measured by the Resilience Scale for Adolescents (READ) among unaccompanied minors compared to a control group of age- and gender-matched adolescents. Based on the limited existing research [33] we expected unaccompanied minors to report similar resilience factors compared to the control group. A second aim was to examine the association between these resilience factors and protective predictors among unaccompanied minors including gender, amount of support in living situation, contact with family in the country of origin, frequency of contact with the contact person in the child welfare services (CWS), and number of leisure activities. Based on previous literature, we expected that male gender, high support living situation, frequent contact with family in the home country, frequent contact with contact person, and more leisure activities would be associated with higher levels of resilience factors.

## Method

### Participants and procedure

Data stems from the “Pathways to Independence”- study (PTI), a comprehensive cross-sectional study conducted among unaccompanied minors who were granted residence permits and living in Bergen municipality [35]. Data was collected from December 2018 to January 2019. All informants were recruited from the CWS for unaccompanied minors in the municipality, and the CWS also coordinated the data collection. All unaccompanied minors above the age of 15 were invited to complete the electronic survey. Of 101 invited unaccompanied minors, 81 unaccompanied minors between 15 and 20 years old consented to participate, giving a response rate of 80% [35].

The questionnaires used in PTI were developed in cooperation with the CWS for unaccompanied minors in Bergen [35]. The questionnaires were in Norwegian, as most of them did not exist in officially translated and validated versions in the languages spoken by the unaccompanied minors. A pilot study was conducted among older unaccompanied minors previously under the care of the CWS to test the feasibility of the questionnaire. The questions were reformulated and simplified based on feedback from the pilot study.

The survey was conducted in the caseworkers’ office. It took 1.5–3.5 h to complete the entire survey. Six of the respondents needed interpreters. When no interpreter was present, caseworkers were available for questions and support. They were also available for follow-up when needed.

The responses from PTI were compared to a matched control group from the youth@hordaland survey. Youth@hordaland is a population-based study conducted in Hordaland in the spring of 2012, where all adolescents born between 1993 and 1995 were invited to participate. For school attendees, information about the study and a link to participate was sent by SMS and to their school email address. Those not attending school received information by post to their home address. During the data collection period, the adolescents could respond at their convenience, and the schools allocated one school hour to complete the questionnaire. It took about 45 min to complete the survey. A total of 10,257 adolescents consented electronically to participate, yielding a participation rate of 53%. Of these, 9596 respondents completed the READ and formed the sample the controls were drawn from. Each respondent from PTI was matched with four randomly selected adolescents from youth@hordaland (*n* = 324). The control group was matched by age and gender, due to the skewed distribution of gender in the sample from PTI (17.3% female). The eligible age range of matches was set to ± 1.1 years to achieve a 1:4 ratio. The youth@hordaland study was considered a good match for the unaccompanied minors because it consists of a well-defined cohort of adolescents in approximately the same age range and located in the same county as the unaccompanied minors.

### Ethics

Both the PTI project (2018/966) and the youth@hordaland study (2012/1467) were approved by the Regional Committee for Medical and Health Research Ethics of Western Norway and conducted in accordance with recommendations from the Norwegian Data Protection Services. All participants aged 16 or older consented to participation, and for unaccompanied minors aged 15, a legal guardian also gave assent. Both studies were voluntary, and the respondents could withdraw from participation at any time.

### Instruments

#### Sociodemographic information

The unaccompanied minors reported age, years since arrival, gender, and country of origin in PTI. Years since arrival were calculated by subtracting the age at participation by age at arrival.

The gender and age of the adolescents in youth@hordaland were retrieved from the personal identity number in the Norwegian National Population Register. Parental education level and perceived economic well-being were reported by the adolescents. Parental educational level was divided into *basic* (elementary school), *intermediate* (high school), *higher* (college/university), and *unknown* education levels. Perceived economic well-being was reported as the family’s economic circumstances being either: “poorer than others”, “equal to others”, or “better than others”.

#### Predictor variables

The following predictor variables were included from PTI: gender, living situation, frequency of contact with family in the home country, frequency of contact with the contact person in the CWS, and the number of leisure activities. The unaccompanied minors’ living situation was dichotomized based on how much support it entailed. The *more supportive* category included those living in foster care, shared accommodation, or in an institution. The *less supportive* category included those living in a host family or municipal/private housing. The frequency of contact with family in the home country was divided into two groups, one for *contact weekly or more often*, and one for *contact monthly or less frequently.* Contact with the contact person in the CWS was also divided into two groups, one for *contact weekly or more often*, and one for *contact 2–3 times a month or less frequently.* The number of leisure activities measured how many activities they attend regularly, with options of none or a list of activities where they could cross all the activities they attend. The scores were combined in a sum score.

#### Outcome variables

READ was used to measure self-reported resilience factors [36]. READ consists of 28 statements, where the responses are rated on a 5-point Likert scale from “completely disagree” (1) to “completely agree” (5). Originally, READ is divided into five factors: Personal Competence, Social Competence, Family Cohesion, Social Resources, and Structured Style. The PTI project included the items from four of the five READ factors while leaving out the items measuring Family Cohesion as this scale was considered less relevant in the sample of unaccompanied minors [35].

The factor structure of this abbreviated version of the READ was investigated in the PTI sample using confirmatory factor analysis (CFA) and the maximum likelihood estimator with robust standard errors (MLR). Model fit was assessed using the Tucker-Lewis index (TLI), the comparative fit index (CFI), and the root mean square error of approximation (RMSEA). TLI and CFI values greater than 0.90 indicate an acceptable fit to the data, while values greater than 0.95 indicate an excellent fit [37, 38]. Regarding RMSEA, values below 0.080 are considered acceptable [38]. The original READ model yielded a relatively poor model fit in terms of the CFI, TLI, and RMSEA in the CFA (*x*^2^(203) = 411.812, *p* < 0.001, CFI = 0.861, TLI = 0.841, RMSEA = 0.105).

As the READ has previously been validated in the youth@hordaland study [39], suggesting a modified five-factor structure where items from Personal Competence and Structured Style were reorganized into Goal Orientation and Self-Confidence, a CFA was performed on the revised factor structure also in the PTI-sample. Investigating the modified factors yielded an acceptable model fit in terms of the CFI, the TLI, and the RMSEA (x^2^(136) = 1148.302, *p* < 0.001, CFI = 0.950, TLI = 0.940, RMSEA = 0.074). As the modified version yielded a better model fit, these factors were used in the analyses. The Cronbach’s alpha was 0.85 for the factors Goal Orientation, Self-Confidence, and Social Competence and 0.87 for Social Support. Across the factors, a higher score indicates more resilience factors.

When summarizing items into the four READ factors, participants who responded to more than half of the items in the subscales were included. For analyses on item level, missing responses were not included. In the unaccompanied minor sample, 11 adolescents (13.4%) had missing on one or more items, and in the matched control group, 3 adolescents (0.9%) had missing responses on one or more items.

### Statistical analyses

All analyses were performed using STATA SE version 17.0. Figures were made in R version 4.2.2 for Windows using ggplot2 [40].

To compare self-reported resilience factors in unaccompanied minors and a matched control group of adolescents, Welch t tests were conducted on both item and subscale levels due to different variances and *n* in the groups.

Associations between the resilience factors and gender, living situation, contact with family in the country of origin, frequency of contact with the contact person in CWS, and the number of leisure activities were investigated in bivariate regression analyses, only among the unaccompanied minors. To ease comparison between the different READ factors, the scores were standardized with a mean of 0 and a standard deviation (SD) of 1.

Effect sizes from the Welch t tests were calculated using Cohen’s d. Cohen’s d effect sizes were interpreted according to Cohen [41], where 0.2 equals a small effect, 0.5 equals a medium effect and 0.8 equals a large effect. The *p* values are presented at a 95% significance level.

## Results

The demographic characteristics are presented in Table 1. Among the unaccompanied minors, the most common background was Afghan (46.9%), followed by Eritrean, Syrian, and Somali. The most common living situation was municipal/private housing (46.9%). Only 33% of the unaccompanied minors stayed in frequent contact with family in the country of origin, and 32% had weekly or more often contact with their contact person in CWS. The unaccompanied minors reported on average two leisure activities. In the matched control group, only 6.8% were foreign-born, and most of the youth had parents with intermediate or higher education (34.3% and 40.4%, respectively). Most of the adolescents from the youth@hordaland perceived their family’s economic well-being as “equal to others” (65.8%).

**Table 1:** Sociodemographic characteristics of the participants in the Pathways to Independence study (*N* = 81) and a matched control group from the youth@hordaland study (*N* = 324)

	Unaccompanied minors (*N* = 81)	Matched control group (y@h) (*N* = 324)
	Data presented as *n* (%) or Mean (SD) [range] values	
Age	18.0 (1.3) [15–20]	17.9 (1.0) [16–19]
Female gender	14 (17.3)	56 (17.3)
Time since arrival (years)	3.5 (2.2)	–
Country of origin		
Afghanistan	38 (46.9)	–
Eritrea	14 (17.3)	–
Syria	14 (17.3)	–
Somalia	7 (8.6)	–
Other^a^	8 (9.9)	–
Living situation		
Municipal/private housing	38 (46.9)	
Host family	14 (17.3)	
Shared accommodation	13 (16.1)	
Foster care	8 (9.9)	
Institution	8 (9.9)	
Contact with family		
Weekly or more often	27 (33.3)	
Monthly or less frequent	54 (66.7)	
Contact with contact person		
Weekly or more often	26 (32.1)	
2–3 times a month or less frequent	55 (67.9)	
Leisure activities	2 (1.4) [0–5]	
Foreign-born	–	21 (6.5)
Maternal education		
Basic	–	21 (6.5)
Intermediate	–	107 (33.1)
High	–	123 (38.1)
Unknown	–	72 (22.3)
Paternal education		
Basic	–	40 (12.4)
Intermediate	–	105 (32.5)
High	–	107 (33.1)
Unknown	–	71 (22.0)
Perceived economic wellbeing		
Worse than others	–	22 (6.9)
Equal to others	–	200 (62.9)
Better than others	–	96 (30.2)

^a^Ethiopia, Palestine, Congo, and Sudan

Matched control group (y@h): matched control group from the youth@hordaland study; ethnicity y@h reference: Norwegian

Unaccompanied minors reported fewer resilience factors on all subscales compared to the control group. For Self-Confidence, the difference was non-significant (see Table 2). Social Support had the largest group difference with a medium effect size.

**Table 2:** Mean (SD) scores on the READ subscales in 81 unaccompanied refugee minors compared to a matched control group

Variable	Mean score (SD) of the unaccompanied minors	Mean score (SD) of y@h	Cohen’s *d*	*p *value
Goal Orientation	3.53 (1.19)	3.84 (0.77)	0.36	0.03
Self-Confidence	3.72 (1.01)	3.84 (0.87)	0.13	0.33
Social Competence	3.61 (1.11)	3.96 (0.83)	0.39	0.01
Social Support	3.81 (1.13)	4.38 (0.73)	0.70	< 0.001

Results from Welch *t* tests

**Fig. 1:**
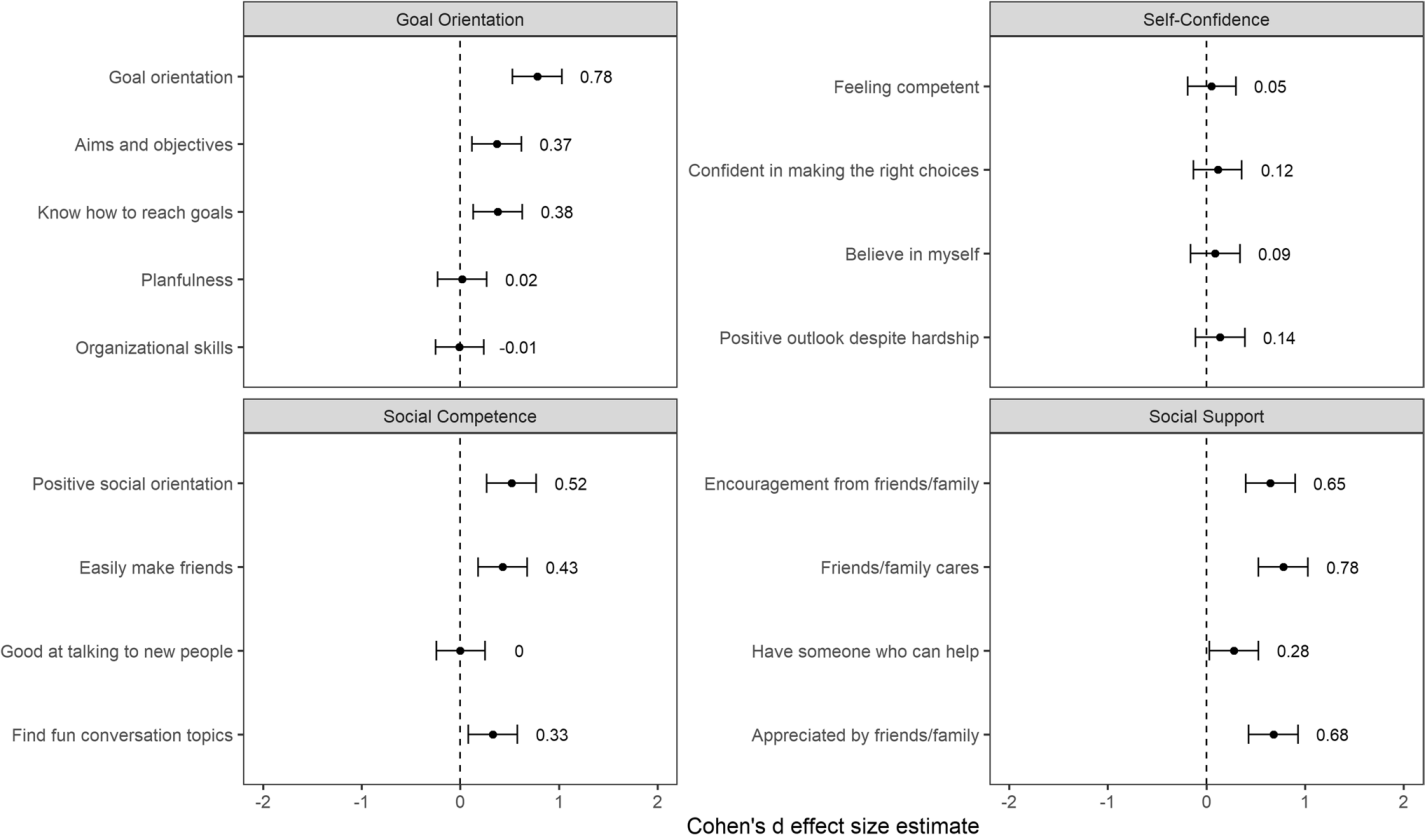
Standardized mean difference in scores on READ items between unaccompanied minors and matched control group. Note: Cohen’s d with 95% CI. There is no difference in the scores of the reference group of unaccompanied minors and the matched control group at 0, positive deviations indicate higher scores in the matched control group while negative scores indicate lower scores in the matched control group

Figure 1 shows the difference in scores on each READ item between unaccompanied minors and the control group. In the Social Support subscale, the unaccompanied minors scored lower on all four items. In the subscale Goal Orientation, unaccompanied minors had lower scores than the control group on three of the five items. Regarding Social Competence, unaccompanied minors scored lower on three of four items. There were no differences between the groups on any of the items in the Self-Confidence subscale.

**Fig. 2:**
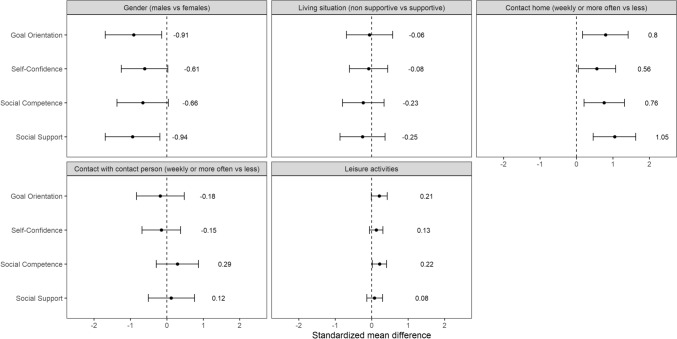
Associations between known resilience factors and READ subscale scores in 81 unaccompanied minors. Note: Standardized mean differences with 95% CI

Figure 2 presents the associations between READ subscales and characteristics previously identified as protective among unaccompanied minors. Male gender was associated with lower scores on all READ subscales but was only significant for Goal Orientation (SMD = − 0.91, 95% CI = − 1.69, − 0.14) and Social Support (SMD = − 0.94, 95% CI = − 1.69, − 0.19). SMD for Self-Confidence and Social Competence were − 0.61 (95% CI = − 1.25, 0.03) and − 0.66 (95% CI = − 1.37, 0.40), respectively. Having frequent contact with family in home country was positively and significantly associated with all subscales, with SMDs ranging from 0.56 for Self-Confidence to 1.05 for Social Support. Supportive living situation and frequency of contact with the contact person in the CWS showed no significant associations with the READ subscales. The number of leisure activities was significantly associated with Social Competence with an SMD of 0.22, but not with the remaining factors.

## Discussion

This is the first study among unaccompanied minors examining resilience using a validated measure and an age- and gender-matched control. Overall, the unaccompanied minors reported fewer resilience factors than a matched control group from a Norwegian general population, except for Self-Confidence. Females scored higher than males on all the subscales and the unaccompanied minors with frequent contact with family in their home country scored higher than the unaccompanied minors with less frequent contact. Few differences were detected for living situation, frequency of contact with the contact person, and the number of leisure activities.

Unaccompanied minors reported fewer resilience factors on Goal Orientation, Social Competence, and Social Support with small to medium effect sizes. Due to the novelty of this study, there are no studies available for direct comparison. Only one study compared the resilience factors reported by unaccompanied minors to a control group [33]. However, the control group consisted of 1451 adolescents from 11 different countries who faced different forms of adversity in childhood. Thus, due to the heterogeneity and the adversity the control group has faced, findings are not directly comparable. Notably, there was no significant difference between the unaccompanied minors and the matched control group in the Self-Confidence subscale.

Male unaccompanied minors reported fewer resilience factors than females on all subscales, but the gender difference was not significant for Self-Confidence and Social Competence. This is in line with a study where females reported higher scores on Social Support from co-ethnic friends [21]. Still, it contrasts with the well-established gender difference in mental health problems, where females report more mental health problems (suggesting less protection) [5, 10–12, 17, 18, 20–24]. Mental health problems may develop differently from protective factors, where mental health problems among females can be related to experiences more traumatic events, such as sexual abuse, rape, or forced prostitution during migration [19]. Despite the low number of girls, our study indicates that female unaccompanied minors have more resilience factors in certain life domains than males. There are likely similar gender differences for Self-Confidence and Social Competence, though these were not significant, as the SMD was about 0.6 for both.

Frequent contact with family in the home country was associated with higher levels on all resilience factors, in line with the literature on the impact of family support on mental health outcomes [13, 17, 21, 29, 30]. In contrast, one study found no association between contact with family and social support from others outside the family [21]. However, they did not include frequency of contact. Our finding corresponds with the resilience literature where the most important protective factor for children and adolescents is support from a safe adult, most often a parent [8]. Of note, the frequency of contact could also be related to other factors such as lack of family members to contact because of war or other traumatic events. Results should therefore be interpreted with caution.

In contrast to previous studies [12, 17, 18, 22, 25, 27, 29], there was no association between the amount of support in the living situation and the resilience factors. In previous studies, high support living arrangements such as foster care were protective in terms of mental health outcomes [12, 18, 19, 22, 27]. The present findings may be explained by our categorization, where several different living arrangements were combined and not investigated separately. Contact with the contact person in the CWS and leisure activities were included as proxies for social support, which is important in fostering mental health among unaccompanied minors [14, 17, 21, 30]. Contrary to our hypothesis, frequency of contact with the CWS was not associated with the resilience factors. The number of leisure activities was only associated with Social Competence, while the SMDs for the remaining factors were similar in size, although non-significant, indicating a power issue. Our findings indicate that more leisure activities can bolster social competence among unaccompanied minors, partly in line with a study where participating in activities such as sports was associated with reduced mental health symptoms [14].

There has been a lack of research among unaccompanied minors that use established resilience measures, which is important as more knowledge about resilience factors will contribute to the understanding of mechanisms underlying positive development [33, 42]. Bronstein, et al. [27] pointed out the importance of understanding the protective mechanisms for unaccompanied minors through applying a resilience framework which will provide knowledge about the appropriate support given specific risk exposures. Shifting the focus from symptoms only, to coping mechanisms and resilience is important [15], and developing standardized measurements is key to being able to compare and share results across cultures and contexts [43].

### Strengths and limitations

#### Strengths

The main strength is the data from a hard-to-reach sample of participants. The use of an age- and gender-matched control group is also considered a strength because most research on unaccompanied minors either uses other high-risk groups as a control group or excludes control groups entirely.

#### Limitations

First, the questionnaires used in PTI were all in Norwegian. Even though the study took precautions, there is a possibility for misunderstanding when filling out the questionnaires. The presence of child welfare workers and the location could also impact the responses. All the unaccompanied minors in our sample were settled in Bergen Municipality. According to the Norwegian Directorate of Integration and Diversity, settlement is based on the needs of the minor and the capacity of the municipalities. Thus, we have no indications that our sample differs from the unaccompanied minors settled in other municipalities [44] and the sample is representative of adolescent unaccompanied minors in Norway in terms of origin, age, and gender [45].

Second, the small sample of unaccompanied minors results in limited statistical power to detect differences within the group of unaccompanied minors, and estimates should be interpreted with caution (as confirmed by wide confidence intervals). The results presented indicate a need for studies with larger samples in the future to support and nuance our findings.

Third, there is a high number of missing (13.4%) among the unaccompanied minors.

Fourth, this is a descriptive, cross-sectional study and does not allow us to draw any causative conclusions. Also, our findings could be influenced by factors that we are unable to control for. Further, the directionality of the results is not certain, the associations between the predictor variables and resilience factors could be explained by the adolescents reporting more resilience factors doing more positive things, such as being in contact with family in their home country or that the more socially competent unaccompanied minors tend to be involved in more leisure activities.

Fifth, the PTI and the youth@hordaland study were conducted approximately 7 years apart. We cannot exclude the possibility that the different time frames might impact the results.

## Conclusion and implications

In the current study, unaccompanied minors reported fewer resilience factors compared to Norwegian adolescents. This indicates that they may have different needs compared to other adolescents, which is important information for stakeholders and authorities. The fewer resilience factors among the male unaccompanied minors in our sample may indicate a need for greater support compared to female unaccompanied minors, especially concerning self-confidence and social competence. Our findings thus indicate the importance of customized child welfare measures and support according to gender, even as there is a need to examine gender differences in larger samples. Bolstering protective factors and competence could for instance be included in the action plans the CWS workers establish with the minors. The results also indicate the importance of leisure activities for social competence, suggesting that motivating unaccompanied minors to attend activities outside of school and facilitate desired activities are called for. Further, frequent contact with family in the home country was associated with higher scores on all the resilience factors, demonstrating the pivotal role that family plays despite long distances. Facilitating contact with the biological family for unaccompanied minors who express a desire for it, could be an important measure for healthy development when settling in the host country. Our results also underline the importance of an open dialogue between the minor and the CWS workers on the topic of the family in the home country. The CWS could further support and facilitate this communication through digital communication tools and help resolve conflicts that hinder such contact.

## Data Availability

Data are available upon request.
